# Chemotherapy-Induced, Broadly Reactive Autoantibodies in a Colon Cancer Patient

**DOI:** 10.7759/cureus.31954

**Published:** 2022-11-28

**Authors:** Felix Broecker, Elena Shanin, Nikolay Lysov, Vadim Shanin

**Affiliations:** 1 Diagnostics and Cancer Vaccines, OncoVax Theranostics Co.Ltd., Burgas, BGR; 2 Diagnostics, Reaviz Burgas Co. Ltd., Burgas, BGR

**Keywords:** agglutination assay, rapid diagnostic, igm, autoimmunity, toxicity, chemotherapy, adenocarcinoma, colon cancer

## Abstract

The link between cancer and autoimmunity is well-established. For example, increased levels of autoantibodies are frequently found in cancer patients, and autoimmune diseases are linked to an increased risk for certain neoplasms. However, the extent to which chemotherapy induces autoimmune reactions remains largely elusive. Here, we quantified immunoglobulin M (IgM) responses to various human tissues and the patient’s tumor before and during adjuvanted chemotherapy (seven cycles of the FOLFIRI regimen (folinic acid/fluorouracil/irinotecan) plus cetuximab) of a patient with metastasized colon cancer. IgM levels against all investigated tissues increased shortly after the first cycle and were further boosted by cycles two and three. Autoimmune responses then decreased during cycles four to seven but remained above baseline levels for most tissues. Our findings suggest that chemotherapy can induce broadly reactive autoimmune responses. Monitoring self-reactive IgM responses during treatment may help alleviate autoimmunity-related adverse events.

## Introduction

Malignant disease is a known cause of autoimmune responses that include the generation of autoantibodies [1]. Cancer-induced autoimmunity has been linked to the development of various autoimmune disorders such as rheumatic diseases [1,2], paraneoplastic cerebellar degeneration [3], and others [4]. These disorders can at least partially be caused by autoantibodies raised against tumor antigens that are also expressed in healthy tissues. These antibodies can, for example, be induced by intracellular antigens released from apoptotic tumor cells [5] or by mutated proteins that elicit antibodies cross-reacting to the non-mutated counterparts. For instance, antibodies against a tumor-specific mutant of RNA polymerase III subunit (RPC1) were shown to cross-react with normal RPC1, thereby contributing to the autoimmune disease scleroderma [6,7]. To date, over 100 autoantibody specificities have been identified in various neoplastic diseases, with potential uses in diagnosis and treatment [8]. Cancer-induced autoantibodies can be of the immunoglobulin M (IgM) (primary response), IgG (secondary response), and IgA (mucosal) classes [9,10]. Thus, cancer-induced, self-reactive IgM responses can undergo affinity maturation and generate immunological memory.

In contrast, little is known about the effect of chemotherapy on autoimmune responses. Chemotherapy-induced cell death leads to the release of tumor antigens from dying tumor cells along with damage-associated molecular pattern molecules (DAMPs), which bind receptors on immune cells, stimulating immune responses [11]. However, only a few associations between chemotherapy and autoimmunity have been reported. A recent case report described a patient with chemotherapy-induced encephalitis [12]. Furthermore, hemophagocytic lymphohistiocytosis [13] and arthropathy [14] have been linked to chemotherapy, with evidence for the involvement of autoantibodies in arthropathy [14]. Cetuximab (anti-eGFR (estimated glomerular filtration rate) monoclonal antibody) has been shown to deplete regulatory T (Treg) cells [15], possibly facilitating the activation of autoantigen-specific B cells. A comprehensive study on the autoimmunity induced by chemotherapy and monoclonal antibody therapy, however, is lacking. Therefore, we determined here the autoimmune reaction of a patient with metastasized colon cancer following combined adjuvant chemotherapy and monoclonal antibody therapy. To this end, we used an agglutination assay to quantitate IgM antibodies (reflecting the primary autoimmune response) against various human organs and the patient’s tumor. IgM responses were observed before and during seven cycles of treatment with folinic acid/fluorouracil/irinotecan (FOLFIRI) combined with cetuximab. We found that the treatment was associated with a severe increase of IgM reactive to all investigated tissues, especially during the first three treatment cycles. Although we observed a decline in IgM levels during the following cycles, IgM against most organs remained above baseline until after the seventh cycle. Our data support that chemotherapy can trigger autoimmune reactions against various human tissue antigens. Monitoring of the IgM responses may help prevent or ameliorate immune-related adverse events in response to chemotherapy.

## Case presentation

A 55-year-old woman was diagnosed with cancer of the sigmoid colon via a colonoscopy that was performed because of reported periodic bleeding during defecation. There was no evidence of dissemination of the tumor. A sigmoidectomy was performed. Histological examination revealed a moderately differentiated colorectal adenocarcinoma penetrating adipose tissue, with extensive lymphovascular invasion. The patient received eight cycles of chemotherapy (XELOX regimen (capecitabine plus oxaliplatin)). Positron emission tomography-computed tomography (PET-CT) and magnetic resonance imaging (MRI) scans detected no distant metastases.

About one year after the initial diagnosis, abdominal ultrasound and a PET-CT scan revealed ovarian cancer of potential metastatic origin. Further suspected metastases were identified in the lung as well as an axillary lymph node. The patient underwent extirpation of the uterus with appendages and the omentum. Histology of the removed organs revealed a moderately differentiated adenocarcinoma of the left ovary, likely of metastatic origin. Immunohistochemistry revealed that the phenotype of the tumor was consistent with colorectal carcinoma. A follow-up CT scan showed metastasis in the left lung. Consequently, the patient received radiotherapy treatment for this metastasis, which was shown to be successful by following PET-CT scans.

About nine months later, a PET-CT scan revealed a new hypermetabolic nodule in the left lung and hypermetabolic lymphadenopathy in a subcarinal lymph node, most likely of neoplastic nature. Hypermetabolic foci were identified in the rectus abdominis muscle and the right iliac region. A total biopsy of the metastatic focus of the anterior abdominal wall with partial resection of the rectus abdominis muscle on the left was performed. Histological findings were consistent with adenocarcinoma metastasis.

**Figure 1:**
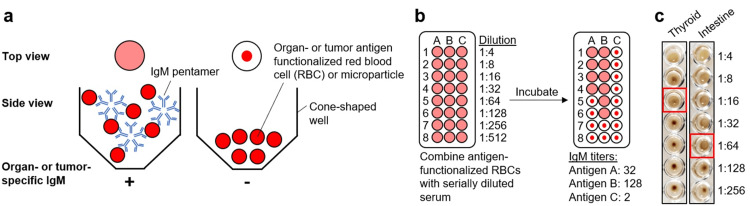
Principle of the assay for IgM quantification (a) Red blood cells (RBCs) or microparticles are surface-functionalized with antigens extracted from organs or tumor tissue. In the presence of antigen-specific immunoglobulin M (IgM) (left), the RBCs/microparticles are agglutinated (formation of a lattice), visible as a diffuse light red suspension. In the presence of antigen-specific IgM (right), the RBCs/microparticles are not agglutinated and sink to the bottom of the cone-shaped well, visible as a distinct dark red dot. (b) Titration of serum samples to determine IgM titers against different antigens (A, B, and C). The IgM titer is defined as the highest serum dilution factor for which agglutination is observed. (c) Representative images of agglutination assays using the patient’s serum after the third cycle of chemotherapy to the indicated organs. The wells representing the inferred IgM titers are highlighted by red squares.

The patient received seven cycles of chemotherapeutic treatment with the FOLFIRI regimen plus cetuximab over a time period of about three months. PET-CT evaluation confirmed the stabilization of the patient. To monitor the patient’s health status and the autoimmune response to chemotherapy, we quantified self-reactive IgM responses at organ-level resolution using an investigational diagnostic approach. Briefly, soluble components of various human organ homogenates, and of the patient’s tumor tissue, were immobilized on the surface of goat red blood cells (RBCs). The human antigen-functionalized RBCs were incubated with serial dilutions of the patient’s serum, whereby organ-specific IgM, due to its oligomeric nature, cross-links the RBCs (Figure 1a). The serum-RBC mixture is incubated for about one hour. Then, similar to a standard hemagglutination assay, the readout relies on the differentiation of diffuse wells vs. wells with RBCs settled down at the bottom of a U- or V-bottom-shaped 96-well plate due to gravity. The serial serum dilutions allow for quantifying organ-specific IgM titers expressed as serum dilution factors (Figure 1b). Images of representative agglutination assays using the patient’s serum are shown in Figure 1c.

**Figure 2:**
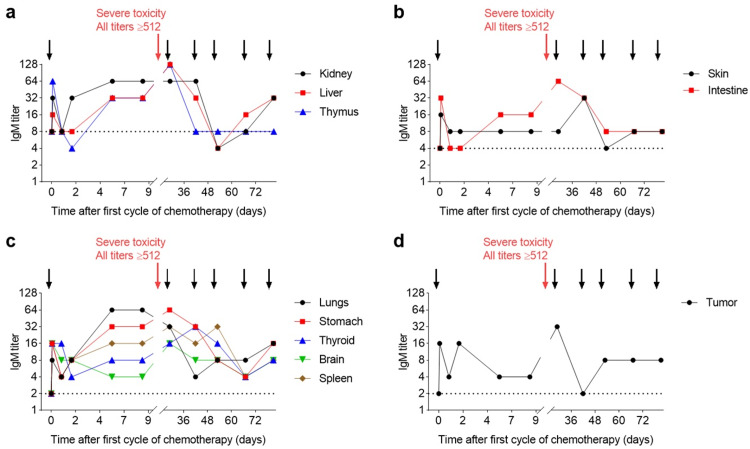
IgM titers to various organs and the patient’s tumor before and during chemotherapy In all panels, arrows indicate the time points of chemotherapy (FOLFIRI plus cetuximab) administrations, whereby the second administration that induced severe acute toxicity is highlighted by a red arrow. Titers immediately following the second cycle were ≥512 for all tissues (not shown in the graphs). Panel (a) shows organs with a relatively high baseline titer, (b) and (c) those with an intermediate and low baseline titer, respectively. Panel (d) shows IgM titers against the patient’s tumor. For samples with no detectable hemagglutination at the lowest serum dilution (4-fold), the IgM titer was defined as 2. The dotted horizontal lines indicate baseline titers observed before the first cycle of chemotherapy. IgM: immunoglobulin M; FOLFIRI: folinic acid/fluorouracil/irinotecan

For all organs and the tumor tissue, we observed a fast initial increase in IgM levels (four- to eightfold increases compared to baseline). The following day the titers decreased, but then increased again and stabilized at above baseline at six to nine days post the first cycle of chemotherapy. IgM titers to the lungs (titer of 64, a 32-fold increase compared to baseline) and stomach (titer of 32, a 16-fold increase) were especially high (Figure 2c). During the administration of the second cycle, the patient experienced severe acute toxicity. The cycle was successfully completed by reducing the infusion rate to control the toxic effect. IgM titers to all organs immediately after the second cycle were ≥512; the final serum dilution factor tested. High titers of self-reactive IgM are predictive of a severe and potentially fatal autoimmune response. Therefore, immunosuppressive treatment was initiated comprising i.v. administration of Ringer-Locke solution, glucose, hydroxyethyl starch, and IgM-enriched immunoglobulins (Pentaglobin), as well as glucocorticoids (Prednisolone), anti-histamines (H1 and H2 histamine blockers), and heparin. In the following, the patient’s overall condition stabilized. After the third cycle of chemotherapy, titers to all organs decreased relative to post the second cycle but remained relatively high (32 or 64) for the liver, thymus (Figure 2a), intestine (Figure 2b), lungs, stomach, spleen (Figure 2c), and the tumor tissue (Figure 2d). Additional cycles did not lead to further increases in IgM titers but rather decreases. After the final seventh cycle, IgM titers were two- to eight-fold above baseline levels for the tumor and all organs except for the thymus, which returned to baseline titers.

Shortly after the seventh cycle of chemotherapy, the patient was hospitalized with symptoms of acute respiratory infection. A polymerase chain reaction (PCR) test for coronavirus disease 2019 (COVID-19) was positive. The patient passed away due to severe pneumonia about three weeks later.

## Discussion

We observed an increase in autoreactive and cancer-specific IgM antibodies following chemotherapy with the FOLFIRI regimen plus cetuximab. Although this is merely a correlation, the steep and substantial increase in IgM titers observed immediately after the second cycle provided evidence that chemotherapy had a causative effect on elevated autoantibodies in this case. Moreover, chemotherapeutic drugs, in general, exert cytotoxicity and therefore can damage cells of vital organs. This may induce autoimmune responses through various mechanisms. For example, cell death can lead to the release of autoantigens along with DAMPs, which can stimulate immune responses [11]. Furthermore, cetuximab-induced depletion of Treg cells [15] may have facilitated the activation of autoantigen-specific IgM-producing B cells. Another mechanism that has been demonstrated experimentally in the mouse model is that tissue damage can lead to exposure of neoepitopes that are recognized by circulating autoreactive IgM antibodies [16]. This can trigger the expansion of IgM-producing B cells [16,17]. Induction of autoreactive adaptive immune responses and expansion of self-reactive immunoglobulins after physical injury (such as blunt abdominal trauma or burn wounds) likely involves uptake of complement-opsonized apoptotic cells by dendritic cells (reviewed in [18]). It remains to be shown if the above-mentioned mechanisms also apply to chemotherapy-induced tissue damage and the autoreactive IgM responses, as observed in this study. The high titers of autoimmune IgMs to all investigated organs following the severe toxicity-inducing second cycle of chemotherapy/cetuximab may have been reflective of an autoimmune storm, a condition that has previously been reported following pembrolizumab (anti-PD1) treatment of a lung cancer patient [19]. In the latter (fatal) case, pembrolizumab induced an inflammatory state of various organs (thyroiditis, myocarditis, pneumonitis), along with elevated levels of various autoimmune antibodies, including those against thyroglobulin, thyroid peroxidase, mitochondria, acetylcholine receptor, striated muscle, and others. Monitoring the autoimmune status to different organs during cancer treatment, as in the herein described case, may help timely initiate measures to mitigate therapy-induced toxicity before the onset of symptoms. More generally, autoreactive antibodies have been shown to expand, for example, in patients with autoimmune diseases [14], following physical injury [16,17] or during COVID-19 [20], among others. Early and rapid diagnosis of autoantibody responses at tissue-level resolution may lead to a better prognosis for these patients as well.

## Conclusions

This study concluded that self-reactive IgM against various organs can be induced by chemotherapy. An experimental agglutination-based assay was able to quickly quantitate these IgM responses at organ-level specificity. Monitoring IgM responses may improve the prognosis of patients with cancer or other diseases associated with increased autoantibody levels.
